# Necropolitics of Death in Neurodegeneration

**DOI:** 10.1007/s11013-024-09855-7

**Published:** 2024-04-23

**Authors:** T. de la Rosa, E. Berrocoso, F. A. Scorza

**Affiliations:** 1 Neurology Department, Escola Paulista de Medicina Universidade Federal de São Paulo, São Paulo, Brazil; 2 Neuroscience Department, Universidad de Cádiz, Plaza del Falla 9, Facultad de Medicina 3 Planta, 11003 Cádiz, Spain; 3 Instituto de Investigación e Innovación Biomédica de Cádiz (INiBICA), Cádiz, Spain; 4 Centro de Investigación Biomédica en Red en Salud Mental (CIBERSAM), Instituto de Salud Carlos III, Madrid, Spain

**Keywords:** Necropolitics, Neurodegenerative diseases, Mortality, Death

## Abstract

Neurodegenerative diseases (ND) pose significant challenges for biomedicine in the twenty-first century, particularly considering the global demographic ageing and the subsequent increase in their prevalence. Characterized as progressive, chronic and debilitating, they often result in higher mortality rates compared with the general population. Research agendas and biomedical technologies are shaped by power relations, ultimately affecting patient wellbeing and care. Drawing on the concepts of bio- and necropolitics, introduced by philosophers Foucault and Mbembe, respectively, this perspective examines the interplay between the territoriality and governmentality around demographic ageing, ND and death, focussing on knowledge production as a *dispositif* of power by highlighting the marginal role that the phenomenon of mortality plays in the ND research landscape. We propose a shift into acknowledging the coloniality of knowledge and embracing its situatedness to attain knowledge ‘from death’, understood as an epistemic position from which novel approaches and practices could emerge.

## Introduction

Academic and mediatic discourses are increasingly addressing the interrelated domains of ageing, late life and their accompanying political and social phenomena. An illustrative example is the recent ‘Dying Broke’ feature in The New York Times (Abelson & Rau, 2023), which explores the economic precarity and fragmentation inherent in the long-term care system of the USA, focussing on the experiences of care recipients and caregivers. This state of precarity is marked by constraints within private insurance, underestimations in care utilization, labour shortages, elevated costs associated with assisted living facilities and fiscal prerequisites for government-sponsored care. Ageing is being contextualized within a political space of meaning and power, rendering biomedicine as situated knowledge, permeable to external factors that shape and influence its practical application and consequences. Our intention in this perspective is to expand the analysis of this political space, focusing on a related phenomenon such as neurodegenerative diseases (ND).

ND accounts for one-third of neurological diseases, with Alzheimer’s and Parkinson’s Diseases being the two with the higher prevalence, affecting around 30 million people worldwide (Dorsey, 2018). Ageing is the main risk factor for most ND, and as global life expectancy grows, ND is also expected to see an increasing prevalence in the next decades (Dorsey, 2018; Hou et al., 2019), especially in Western countries showing constrictive demographic pyramids. This could be aggravated by the COVID-19 pandemic, as some evidence points to an association between acute viral infection and neurological symptoms related to neurodegeneration, which could accelerate the prevalence of growth as well as increase the vulnerability and mortality of this population (Chen et al., 2021). Before ND can be treated, they need to be detected. Early diagnosis and treatment are crucial to slowing down the progression and morbidity of ND.

Disabling symptoms such as reduced mobility or dementia are observed in most ND, which often cause a loss of autonomy, affecting the sense of agency and independence, which increases the risk of suffering stigma (Salazar et al., 2019). Patients with ND also have higher mortality ratios when compared with control populations (Dorsey, 2018; Poewe et al., 2017), a trend that has been exacerbated in the last decades (Darweesh et al., 2018; Mackenbach et al., 2014). Thus, ND are not only chronic, degenerative, progressive and debilitating diseases, but also deadly ones. Common causes of death in ND are shared with age-matched healthy population. However, particularities depend on whether the ND affects motor, cognitive or emotional function (Roberson et al., 2005). While much neurodegenerative disease research emphasizes disease progression, early diagnosis and symptom treatment, which undoubtedly impact patients’ wellbeing and significantly improve their quality of life, mortality as a topic is often overlooked (Ritz & Yu, 2000; Scorza et al., 2017). ND, as well as the disability it causes and its mortality, is one of the main challenges faced by biomedicine at the start of the twenty-first century.

Increasing longevity and a shift towards an ageing population invoke among biomedical research a compelling imperative for critical academic inquiry concerning the multifaceted portrayals of ageing, ND and death. Agendas of research funding and development are constructed regarding scientific discoveries, following theoretical and technological trends to deliver the best care for patients. However, they are also shaped by complex social and political interplays which sometimes reflect social injustices and stereotypes placed on determinate groups. Medicine has conquered the political right to use and commodify the human body and its parts (Lock & Nguyen, 2018; Rose, 2006), becoming the sovereign guardian of bodies during health, illness and death. The authority vested in the field to exert influence over the configuration and perpetuation of ND could serve as a pertinent illustration. Instances such as the belief that hysteria was exclusively manifested in women, the assertion that enslaved black bodies experienced less pain or many ideas regarding eugenics are examples of medical knowledge as a proxy of racism, colonialism and domination.

Drawing on Mbembe’s concept of necropolitics (Mbembe, 2019), this review aims to put together some issues regarding the insubstantial role mortality and death have in ND research. Our goal is to point out how social and political factors may shape research trends and discourse in a direction that unfortunately undervalues their lives. Necropolitics can shape the approaches and resource allocation in ND, affecting not only the quality of life and their access to treatment and care, but also their sovereignty and political agency in terms of their bodies. ND, as a case study, effectively exemplifies the performative dimension of necropolitics, something well documented in other various contexts. Furthermore, beyond the profound physical consequences of colonial violence on individuals and populations, necropower also has significant epistemic ramifications, including the suppression and devaluation of knowledge beyond the realm of Western hegemonic science.

Medical knowledge and technologies, in response to the challenges posed by the ageing population, manifest at various levels (Macia et al., 2019), all underpinned by the assumption that medicine holds the prerogative to influence and intervene in the governance of life itself. Building upon the epidemiological insights mentioned earlier, our examination focusses on biomedical contexts situated within the Global North, not as originally regarded by Oglesby or Worsley (Worsley, 1977) in geographical and economic terms, but as a sociological and epistemic demarcation (Santos et al., 2018, 2019). Among this Global North, we direct our attention to spaces exhibiting patterns and relations of power akin to those associated with the Global South (Santos, 2007). Without a process of colonization, patients with ND are placed into specific territorialities, appearing within a Global South inside the Global North (Santos, 2019), subject to some of the colonial necropower relations without having been colonized. We are talking about nursing homes and hospitals located in developed and Western countries, where contrasting territorialities and discourses emerge around the death of certain individuals. Our analysis, by being grounded in such a broad category, may inadvertently overlook some nuances encompassing cultural, spiritual and social dimensions that, in turn, may influence the understanding of care and mortality. However, it is not our objective to propose a comprehensive and universally applicable analysis intended to encompass the full spectrum of these diverse contexts. The decolonizing epistemological approach we advocate necessitates the recognition of the diversity of knowledge and meaning of death in ND present around the world, demanding active engagement of voices that are usually silenced. Listening to these voices may facilitate not only the development of grounded and contextually embedded knowledge through scientific methodologies originating in the Global North, but also the adoption of innovative methods and categorizations to engender knowledge collaboratively with and from the Global South.

The objective of this endeavour is to enrich and advance prevailing perspectives in the field of ND by acknowledging the interplay of factors that ultimately govern its social and political space. First, we establish biopower, necropower and territorialization as our grounds and tools for the analysis. Second, our argumentation centres on how these power relations permeate ND as a biomedical category, illustrating how these technologies not only organize and govern life, but also influence death. Finally, we explore the link between power and knowledge and between ND political space and its scientific framework to emphasize the relationship between necropower territorialities and the coloniality of knowledge, so that we may integrate new perspectives and voices in the generation of knowledge to cultivate a renewed ethos of care.

## Necropolitics: Definition and Uses

Since it was first introduced by Kjellen in 1905, the term biopolitics has been reformulated by many political scientists and philosophers, even by one of the most cited philosophers of the twentieth century, Foucault, who first used the term in 1974 (Foucault, 1974) to refer to a new way of power agency, starting between the seventeenth and eighteenth centuries, that situates life in the centre of political and social domination. For Foucault, biopolitics was applied through dispositifs of biopower that controlled every space of life of the individual through behavioural prescriptions (Foucault, 1976a). The theoretical framework of biopolitics and biopower developed by Foucault has been instrumental in elucidating the mechanisms of power exertion over individuals and populations in modern societies. We are not undertaking an exhaustive analysis of the utilization of this concept, but rather outline its fundamental elements in the context of our argument, recognizing that comprehensive examinations of this concept have been thoroughly conducted by others (Bird & Lynch, 2019; Lemke et al., 2011).

Biopower makes a calculus of value for life, developing in a first form focussed on the discipline of the anatomo-politics of the human body as a machine (discipline, schools and factories) and a second form focussed on the biological mechanisms of the human population as a species (public health, birth rate and migration). As a result, in late modernity, humans are governed by a set of rules concerning multiple social fields, ranging from sexual behaviour to food intake, assembling a complex web of power relations in contemporary societies (Foucault, 1976b), highlighting how the state exerts control over individuals and populations, aiming to regulate and optimize life. In pursuing this endeavour, they engender pivotal inquiries about the character and extent of governmental jurisdiction, alongside the boundaries delineating individual autonomy and efficacy within contemporary societies. This unveils the recognition that individuals find themselves intricately entwined with an established framework of regulations, which are not solely enforced by authoritative entities but also perpetuated through their own invocation and subsequent reinforcement via biological, affective and social commitment.

Foucault’s examination of biopower in his lecture ‘Security, Territory, and Population’ (1978) deepens our understanding of its intersection with capitalism and the state. He underscores that a fundamental task of biopower is the safeguarding of the labour force within the population, thereby enhancing the economic value of individuals. These insights concerning the interconnections between biopower and capitalism serve to underscore the manners in which economic and political systems amalgamate and are mutually strengthened, ultimately shaping the priorities and objectives of individuals occupying positions of authority. The state establishes this societal framework through the utilization of three disciplinary modes: legal punitive measures, vigilant supervision and the security apparatus as a dispositif (Foucault 1976a, 1976b, 1980). From this latter point, Foucault makes evident how biopower is not unilaterally imposed from the top down, but rather, it is intricately linked to the realms of discourse, knowledge and truth, all organized within a multidimensional network.

The economy is now invading our private life to such an extent, that now it is essentially life itself. Power, to continue being power, needs to enter these kinds of vital dynamics, and the economic context makes us begin to think about life in terms of value. Our daily life is now marked by an economic macrostructure that organizes it. The commodification of life under neoliberalism has led to the increasing dominance of economic discourses, resulting in the subordination of human life to the logic of the market. Necessary conditions for life, such as food and healthcare, are delivered as market commodities, following a calculus of life throughout its production and delivery. Our own living bodies are now plastic and modifiable to achieve health or extend life, but primarily, to be more productive (Foucault, 1980). This extends from everyday medical practices to the realms of molecular biomedicine, where the construction of conceptions surrounding the essence of life itself becomes manifest (Rose, 2006). Decisions about childbirth, the number of children and the time of death were once reserved to the divine. Only God could decide when and how these events happened. In modernity, biopolitics dispositifs in the form of biomedical technologies are now capable of making these decisions in the name of scientific reason.

Following this line of thought, Cameroonian philosopher Mbembe proposed the term necropolitics to address the social dispositifs controlling the death of individuals (Mbembe, 2019). He introduced this term in response to Foucault’s biopolitics, as he was aware that in colonized societies, even after the end of colonial administration, the value of life was made through controlling of death. Necropolitics include more than the right to kill: they also include the right to revoke life, to expose others to social death, to enslave or other manifestations of political violence. It appears as a theory of the living dead, of those who are not truly alive because they have no sovereignty to make decisions regarding their own body and agency due to social and political coercion. Mbembe’s concept of necropolitics expands on Foucault’s biopolitics, highlighting the importance of the control of death in societies in which power relations are marked by violence.

Necropolitics should not be framed as a recent phenomenon, contrary to liberal, modern and Western values. Instead, we should understand it as the expression of the historically embedded racial, colonial and patriarchal hierarchies constitutive of modernity (Mayblin et al., 2020; Mbembe, 2019). As formulated by William Deming, ‘Every system is perfectly designed to get the results it gets’. Biopolitics and necropolitics are not anomalies or epiphenomena of modern society, but rather, their ultimate expression; an inevitable outcome of the societal and institutional structures of modernity, including the ways in which power is exercised, but also against whom. The philosopher Enrique Dussel points out that Cartesian *ego cogito*, symbolized as liberal, modern and Western culture and science, was preceded by an *ego conquirus,* rooted in historical colonialism and imperialism (Dussel, 1994). The position of the subject who speaks with the arrogance of being the eye of God is made possible by the subject’s geopolitical location as an imperial being.

Modernity was established on the foundation of colonial genocide, an event that normalized death and entrenched the necropolitical order (Mbembe, 2019). This colonial project prioritized the power and interest of the colonizers, resulting in the dehumanization, stigmatization and marginalization of the colonized. According to Fanon, this was made possible through the spatialization of colonial occupation, the compartmentalization of space and the deployment of internal boundaries and limits (Fanon, 1961). The occupied territories were divided into a complex network of borders and isolated cells, creating a clear divider between the colonizers and the colonized territory. In colonizer territories, life is regulated through biopower, whereas in colonized territories, necropower governs life by means of death.

This brings to light how individuals and communities are stripped of their agency and sovereignty, leading to a liminal state of living death which Agamben (1998) called a ‘bare life’. The organizational power of thanatopolitics denies the political dimension of life, making it not worth living and less grievable when lost (Butler, 2009). Mbembe identifies the application of necropolitics with underlying coloniality and class-based racism, present in social phenomena such as immigrant and refugee experience (Mayblin et al., 2020), police brutality (Alves, 2014) or technology application in warfare (Allinson, 2015). If biopower made a calculus of individual life value, necropower operates among the individuals classified as worthless. Both terms should be intertwined, as one appears in the limits and excess of the other, when life is conceived as survival and devoid of political agency. Corporeal and biological security is achieved through obedience to power, where individuals are subject to a kind of erasure (Bargu, 2014). This physical wearing out or ‘slow death’ is a defining condition of their political and historical existence (Berlant, 2015).

Necropolitics has been a recurrent concept in sociological, political and disability studies, achieving renewed attention in the biomedical field after the COVID-19 pandemic (Dall’Alba et al., 2021). This has brought to light the deep-seated colonial power dynamics underlying public health systems (Sandset, 2021), which sometimes perpetuate forms of necropower by denying the right to healthcare (Pele & Riley, 2021). Although the pandemic has brought to the forefront how necropolitical forces can perform in the intersection between health and other forms of oppression, we need to recognize attempts beyond this topic that also analyse medical technologies and discourses using this conceptual toolkit. The necropolitical agenda inherently harbours distinct territorial aspirations (Agamben, 1998), which might span geographical, ontological or epistemic dimensions, giving shape to how life and death are known and governed (Rose, 2006). Comprehending this intricate political space demands acknowledging the territorial dimensions within which ND is situated, by scrutinizing the colonial demarcations that distinguish life from death.

## The Political Space of Ageing and Neurodegeneration

Since the eighteenth century we have been witnessing a global increase in life expectancy, triggered by less child mortality and better hygiene and health care, precipitating ageing populations in Western and developed countries. Despite the progress this event may constitute, it answers partially to the needs of industrialized societies for labour force (Macia et al., 2019). This new demographic configuration was shaped by biopower through specific technologies such as vaccination or contraceptive use (Takeshita, 2012). Parallel necropower dispositifs were also deployed in the Global South to shape populations, such as one-child policies in China (Hesketh et al., 2005) or forced sterilization in Africa (Durojaye, 2018). Both approaches were designed to align with the demands of global capitalism, demonstrating the intricate relationship between demographic changes and power structures.

In the last decades, demographic ageing is increasingly becoming a public health issue as well as an economic and social concern, as ageing populations are also populations at great risk of ND and other comorbidities (Partridge et al., 2018). This adds another layer of complexity, as both phenomena intersect at a pragmatic dimension, but also a political one, raising questions about the allocation of resources, the prioritization of certain types of care over others and decision-making in clinical settings (Fortin et al., 2022). This goes beyond the demographic phenomena, as these populations may be conceptualized within hermetic sociocultural representations (Hillman and Latimer, 2017). The formation of these representations or categories is founded on the identification of shared characteristics that distinguish them from others, in a sort of ‘biopolitical bordering’ (Scheel, 2020), involving ‘the definition of these subjects as intelligible subjects of governance’, something often employed in epidemiology (Führer and Eichner, 2015). Ageing stereotypes are often challenged by positive images of late life, presenting it as vital and independent. However, this may sometimes contrast with some age-related needs, especially those surrounding death (Fleming and Carter, 2022). Representations about ageing are closely linked to those about ND, and in this latter case are intertwined with the more frequent use of biomedical technologies and healthcare.

In ND, representations often emanate from a latent cause, that is, the death of neurons in the nervous system. This neurophysiology conditions the definition of ND and the explanations given to natural phenomena. The authority of necropower to govern and exert control has continuously extended to encompass increasing categories of bodies, including those of women, people with disabilities, the elderly and beyond, thereby evolving into a more overarching jurisdiction over ‘the body’ as a colonial territory (Robertson and Travaglia, 2020, 2022). As seen in other biomedical contexts, there is a reification of distress and symptoms into bodily states, placing the burden of the disease and disability inside patients’ own bodies (de la Rosa and Scorza, 2022; Valverde, 2015). In the age of the ‘neural self’ (Vidal and Ortega, 2017), from the death of neurons in ND also emerge dying individuals, approaching a liminal position between life and death. This is further reinforced by the conceptualization and animal modelling of ND as subtypes of chronic and progressive neural death (Rose and Abi-Rached, 2013). The physiological process represents just one of numerous routes through which a body is subject to necropower. Nonetheless, it stands as a paradigmatic illustration of how the utilization of these technologies circulates back into the knowledge production process, an example of a ‘bio-loop’ (Hacking, 2004).

Individuals are customarily delineated by their productivity, and those who are incapable of engaging in the labour market face devaluation, as exemplified by Harvey’s assertion: ‘Under capitalism, sickness is defined as the inability to work’ (Harvey, 2006). In this sense, patients with ND encounter specific challenges due to age, disability and loss of autonomy, which often translates to a situation of precarity in later life (Abelson and Rau, 2023; Grenier et al., 2017). On the one hand, biopolitical calculus undervalues these lives, concealing them as valid sovereign and autonomous subjects. On the other hand, necropolitics dispositifs operate by positioning them between life and death, depriving them of the autonomy and dignity to decide about their own lives and deaths (Le Theule et al., 2020; Robertson & Travaglia, 2022). Western ideals of self-reliance and individuality, now interiorized and cerebralized as ‘entrepreneur of oneself’ (Martinez-Hernaez, 2020; Vidal & Ortega, 2017), conflict with unproductiveness and need for care. An observational study found a significant association between ‘self-expression’ values held by the population of a determinate country and the rates of mortality from dementia (Mackenbach et al., 2014). The focus of public health on social conditions has been reframed as a matter of individual choices and ‘lifestyle’. This shift overlooks the inherent interdependence and vulnerability of human experience, failing to portray the need for care or assistance as a natural and ordinary aspect of life.

As previously mentioned, ND are usually incapacitating and disabling conditions that require proper care delivery to achieve favourable prognosis and quality of life. Care is a relational, enacted and complex practice (Warren and Sakellariou, 2020), which is often provided by family members, mostly women, who devote themselves to caregiving as a full-time job without any external support (Schulz and Martire, 2004). Caregivers of individuals with ND are often faced with limited resources, access to information or training (Armstrong et al., 2019; Hovland, 2020), which may affect the quality of care they provide (Pick et al., 2019), provoking low levels of satisfaction with medical services among caregivers (Aza et al., 2022). Moreover, research has shown that the burden of caregiving can have negative consequences on the physical and mental health of caregivers, with studies reporting higher rates of depression, anxiety and chronic diseases among caregivers of individuals with ND (Schulz and Sherwood, 2008), sometimes followed by feelings of relief after patient decease (Schulz et al., 2003). In this regard, the social and economic costs associated with caring for individuals with ND can exacerbate its situation (Grenier et al., 2017). Care and support for patients with ND may be subject to rationing or denial on the basis of economic considerations, thereby perpetuating structural inequality. This is itself a form of necropower, which intersects with other forms of oppression such as race, gender or poverty, evidencing how material and social conditions shape care (Dahodwala et al., 2018).

In contrast to pre-industrial societies, in which infant mortality was commonplace and life expectancy was low, death in old age is now viewed as a normal occurrence (O’Neill, 2016; Robertson and Travaglia, 2022; Robinson et al., 2012). Within modern society, the ageing population as a constructed group, and particularly patients with ND, occupy a liminal position in which their mortality is perceived as an expected outcome (Lloyd, 2004). This marginalization of ND patients, as a group who are approaching the end of their lives, is further reinforced by a societal emphasis on youth and productivity, which exacerbates the sense of stigmatization (Salazar et al., 2019). Foucault pointed out how biopower is often self-invocated and reinforced by individuals (Foucault, 1978). Necropower can also be self-invoked, as evidenced by the presence of suicidal and death ideation in newly diagnosed patients, with 42% of individuals exhibiting such thoughts, especially during the initial 90 days following diagnosis (Maxfield et al., 2023). Normalization of death is often reinforced by a medicalized approach to illness that tends to focus on curative treatments and the preservation of physical functioning. A panel of 64 experts voiced their concerns regarding the initiation of palliative care too early in dementia, seemingly implying a lack of optimism or worthiness of life (Van Der Steen et al., 2016). In addition, there is no consistent set of policies specifically dedicated to death and dying itself, and various policies in other areas, such as healthcare and social services, play a role in shaping the conditions surrounding the end of life (Le Theule et al., 2020; Lloyd, 2004). Medicine and healthcare systems are perceived as the authorized governors of death, both in its anticipation and in its aftermath. Prioritizing the extension of life appears to overshadow the acknowledgment of death as an inherent biological reality, often neglecting its broader social, political and spiritual dimensions (Schumacher, 2010).

Empathy is often seen as a crucial element in the provision of quality healthcare. However, the complex and multifaceted nature of ND can make it difficult for healthcare professionals and the rest of society to fully understand and empathize with the experiences of patients and their caregivers (Pick et al., 2019). Necropower dispositif goes along with a suppression of empathy, where the alterity or the other is placed in this liminal position as an ambiguous citizen (Mbembe, 2019). ND lives, deemed as less valuable, are no longer worth our attention, resources or grief (Butler, 2009), perpetuating the privilege of youth, productivity and able-bodiedness, while disregarding the value of those who do not fit into this ideal. This conceives ageing and illness as personal shortcomings. The aesthetic suppression of empathy is an emergent property, latent to this matrix of power relationships, which normalize and obscure the experiences of patients with ND, making their deaths seem unavoidable and ‘on schedule’.

As depicted by Fanon, the colonial project is founded in a fundamental division of territory (Fanon, 1961), as observed in spaces such as barracks, ghettos and police stations. These patterns of territorialization can be observed in the case of ND, where individuals are often isolated in nursing homes, hospitals or their own homes. This spatial confinement often exacerbates the power dynamics underpinning necropower application, and rather than serving as sites of care and support, they become death spaces in which lives are controlled by a network of practices and norms, something sadly proved during COVID-19 at nursing homes, where measures resulted in an excess of mortality (Aalto et al., 2022), disproportionately affecting vulnerable populations (dos Santos et al., 2020; Weech-Maldonado et al., 2021), including patients with ND (Chen et al., 2023; Rutten et al., 2021). This also contributes to heightened feelings of loneliness and abandonment among the survivors (Rodriguez-Rodriguez et al., 2022). Additional instances are discernible in the critical care provided to homeless populations (Jenkins et al., 2023) and the discourses concerning palliative care (Robertson & Travaglia, 2022). ND care spaces are embedded with the violence of these discourses and territorializations, resembling the ‘genocide continuum’ exemplified by Scheper-Hughes (2007) or the ‘peacetime crimes’ of Basaglia (1987).

The political space around ND shows the intricate relationships between life, power and knowledge. For Foucault, relations of power are indissociable from knowledge, while we are all subjects to the truth institutionalized by power (Foucault, 1976b). Research in the field of ND both informs and is informed by power, underscoring the reciprocal relationship between necropolitics and the scientific endeavors within ND.

## Knowledge “From Death”

Death is a universal biological reality shared by every living organism. For humans, it is not merely an opposition to life, but rather a central theme that imbues life with meaning and symbolism. Yet, in Western societies death has frequently been avoided as a subject of discussion despite its profound significance for human existence. In the preface of his book *Death and Mortality in Contemporary Philosophy*, the philosopher Bernard Schumacher says:

“In order to safeguard his happiness, contemporary Western man has contrived to stop thinking at all about death and, more particularly, about his own death, to deny it in a way by maintaining a stony silence with regard to it” (Schumacher, 2010, p. ix).

Neglecting death as a reality helps create necropolitical blind spots where we can find, among other individuals whose lives are deemed as unvaluable, patients with ND (Stamenkovic, 2013). These blind spots are also epistemological ones from which knowledge is not judged valid. The coloniality of knowledge, as described by the sociologist Anibal Quijano, refers to how colonialism has produced and maintained a system of knowledge that privileges the perspectives and interests of the colonizer while silencing the knowledge of the colonized (Quijano, 2000). The consequences of the coloniality of knowledge production and validation have contributed to the perpetuation of systemic inequalities based on race, gender and class. It is organized around institutions and actors situated in the Global North, where the epistemic *ego cogito* and the imperial *ego conquirus* reside (Dussel, 1994), appearing as universal, ahistorical and objective, taking place nowhere in particular. In contrast, knowledge produced by the colonized is categorized as subjective, regional and culturally informed, in definitive, inferior.

Decolonization of knowledge seeks to critically examine this hierarchy behind knowledge production, validation and application, and in doing so, it aims to bring attention to subjects situated in the colonized territory where we can find the ‘Epistemologies from the South’ (Santos et al., 2018, 2019). These subjects, their perspectives and experiences have been historically excluded from knowledge production, as they come from lives that have not been a priori apprehended or recognized as proper lives (Butler, 2009). From the ontology of individuals situated within colonized territories, their recognition as historical and epistemic entities is derived, thus delineating the boundaries of the knowable. Involving these individuals in knowledge production stands to not only enrich the frameworks for understanding ageing, death, and ND care with a more humanistic approach, but also question the prevailing necropolitical status quo. Our proposition for the co-creation of knowledge is far from advocating for a relative or partial understanding due to the demarcation of epistemic boundaries. Instead, it promotes a collaborative process that commences with the proper delineation and recognition of the loci from which knowledge is generated. Situating the knowledge implies understanding it concerning the specific historical, cultural and social contexts in which it is created (Haraway, 1988). Once we uncover this, it may be possible for scientific knowledge to start a horizontal dialogue with the Global South in an ‘Ecology of Knowledge’ (Santos, 2019). This proposal does not render scientific knowledge invalid, nor is it an invitation to reject current treatments in favour of pseudoscientific or unvalidated practices. Instead, it is a recognition of its potential constraints and how they are linked to the historical roots of colonial and capitalist endeavours. The knowledge originating from patients with ND and their communities ought to steer the development of fairer and more comprehensive approaches and care environments. This collaborative contextually situated initiative may also facilitate the reevaluation of death and mortality in the context of ND.

Within this context, we advocate moving beyond the mere contemplation ‘about death’ to a perspective that involves thinking ‘from death’ as an essential epistemic vantage point for effectively addressing the previously discussed concerns. Obviously, this does not mean the production of knowledge from the afterlife, but to regard the epistemic value of knowledge produced by subjects and communities placed in the liminal position between life and death by necropower. Always thinking about ourselves from the perspective of being alive may reflect a survivorship bias. Despite the effort of modern biomedical paradigms to establish humans in a categorical state of health, we are always transitioning between the dichotomies of health and disease and life and death (Schumacher, 2010). Situating ourselves and our knowledge production between these transitioning places could help us restructure new conceptions about death that, in the context of ageing and ND, give meaning to life. Instead of seeing patients with ND as a burden on society, we could view them as valuable contributors to our collective understanding of what it means to be human. Knowledge production ‘from death’ does not imply any kind of glorification of morbidity or the romanticization of suffering, but rather a critical engagement with the existential and social dimensions of mortality, ageing and ND, which are often neglected or pathologized in hegemonic medical discourses. This ecological approach between expert medical knowledge and the one produced ‘from death’ may render valuable insights into the experience of the disease and care. As stated by Sylvie Fortin:

“Expert knowledge is combined with that of patients who benefit from taking part in the production of medical knowledge. This knowledge is no longer limited solely to representations of the disease and the social and cultural experience of suffering, but extends to the recognition of a unique expertise born of the experience of the disease and the experience of care” (Fortin et al., 2022, p. 3).

Examples of these innovative approaches can be found across various disciplines and contexts within biomedical research, in which the traditional research subjects become active agents and are recognized as epistemic beings inside a matrix of power relations and territories. There is a long-standing tradition of psychiatrists exploring the matrix of power surrounding the disorders they treat, revealing how they stem from conflicts within a colonial framework. This tradition traces back to Fanon, who argued that the disorders among Algerians during the war were not attributable to hereditary factors or neural organization but rather to systemic colonial violence (Fanon, 1961). In this line of thought, figures such as Basaglia or Tosquelles crystallized these perspectives into projects of community-based psychiatric care that continue to influence transformative practices nowadays. Efforts and proposals for a renewed psychiatric practice (Rose and Rose, 2023; Rose, 2018) regard the disorders as culturally informed entities (Kirmayer et al., 2017) and colonial dispositifs of power (Mills, 2014; Rivera-Segarra et al., 2022). The area of global and public health has also advocated not only for the right to access healthcare, but also for decolonizing approaches that think of ‘health’ beyond the framework of Global North practices (Adhikari et al., 2023; Pratt and Vries, 2023).

While psychiatry demonstrates, at least partially, a propensity to regard mental disorders as imbued in social and political life, neurology remained predominantly a neurobiological discipline, more closely aligned with the natural sciences and firmly situated within the domain of biomedicine. This might explain the absence of historical references such as ‘critical’, ‘transcultural’ or ‘decolonial’ neurology, beyond the more recent and broad proposal for a ‘critical neuroscience’ (Choudhury and Slaby, 2011). However, this has not prevented some ND researchers from actively engaging in and fostering collaborations between experts, patients and caregivers focussed on the development of integrated and patient-centred care structures (Bloem et al., 2020; Darweesh et al., 2018) and producing knowledge and practices stemming from an open and horizontal dialogue (Hovland, 2020; Pigott et al., 2023).

These may signify a shift towards more inclusive and holistic ND research, where individuals are no longer organized and ruled through value, and their bodies are no longer subject to power dispositifs. For this endeavour, ideas from territories exterior to biomedicine are needed to imagine and construct knowledge in collaboration with and from death as well as from other places of the Global South where the experiences of ND and death directly challenge the hegemonic representations and discourses. We hope that the present perspective has fulfilled its goal of linking and engendering dialogue among ideas that are frequently disparate, elaborating towards renewed epistemic and categorical frameworks that help dissolve the biopolitical and necropolitical matrices of power.

## Conclusions

ND pose a significant challenge for biomedical research in the twenty-first century, given their disabling nature and the high mortality rate they entail. The technological and scientific revolution taking place in neurological research should be accompanied by critical reflections on these scientific advances, especially those reflections concerning the matrix of power relations governing human life and death through the application of bio- and necropower dispositifs. As shown here, the demographic biopolitics that provoked the rise in life expectancy in the first place become necropolitics when the mortality of the ND population is left to the side. New approaches should integrate the biological theoretical framework with the material relations of autonomy and care in end-of-life situations. Leveraging knowledge and insights ‘from death’ could create more inclusive research that prioritizes patient wellbeing and dignity in both life and death.
